# Adult Double-Chambered Right Ventricle Associated With Ventricular Tachycardia and New-Onset Heart Failure

**DOI:** 10.7759/cureus.8122

**Published:** 2020-05-14

**Authors:** Saikrishna Patibandla, Siddhant Trehan, Htoo Kyaw, Kevin Tsai, Sarath Reddy

**Affiliations:** 1 Internal Medicine, Brooklyn Hospital Center, Brooklyn, USA; 2 Cardiology, Brooklyn Hospital Center/Mount Sinai Heart, Brooklyn, USA

**Keywords:** double-chambered right ventricle, ventricular tachycardia, heart failure with reduced ejection fraction

## Abstract

A double-chambered right ventricle is an uncommon form of congenital heart disease that is characterized by the division of the right ventricle into a proximal high-pressure chamber and a distal low-pressure chamber. A 70-year-old male presented to the emergency room from his outpatient doctor’s office with unstable wide complex ventricular tachycardia with right axis deviation. His ventricular tachycardia was terminated using external cardioversion and intravenous amiodarone. He was subsequently found to have new-onset heart failure with a reduced ejection fraction and a right ventricular tract outflow obstruction on transthoracic echocardiography. A diagnosis of the double-chambered right ventricle was made. The patient was offered surgery to fix the anomalous tissue but he refused. He did agree to subcutaneous implantable cardioverter-defibrillator placement and was then discharged home.

## Introduction

Double-chambered right ventricle (DCRV) is an uncommon form of heart disease in which the right ventricle is divided into two different chambers, a proximal high-pressure chamber and a distal low-pressure chamber, and is characterized by anomalous muscular bands (type 1) or marked parietal and septal muscle hypertrophy (type 2) [1]. Type 1 DCRV patients tend to lack associated defects while type 2 DCRV patients tend to have associated defects [2]. DCRV is often discovered in patients at a young age and corrected in their youth but may also present later on in adulthood. We present the case of a patient who had been living with this congenital heart disease for nearly 50 years. He presented with unstable wide complex ventricular tachycardia with right axis deviation and was subsequently found to have heart failure with a reduced ejection fraction and a right ventricular outflow tract (RVOT) obstruction.

## Case presentation

**Figure 1:**
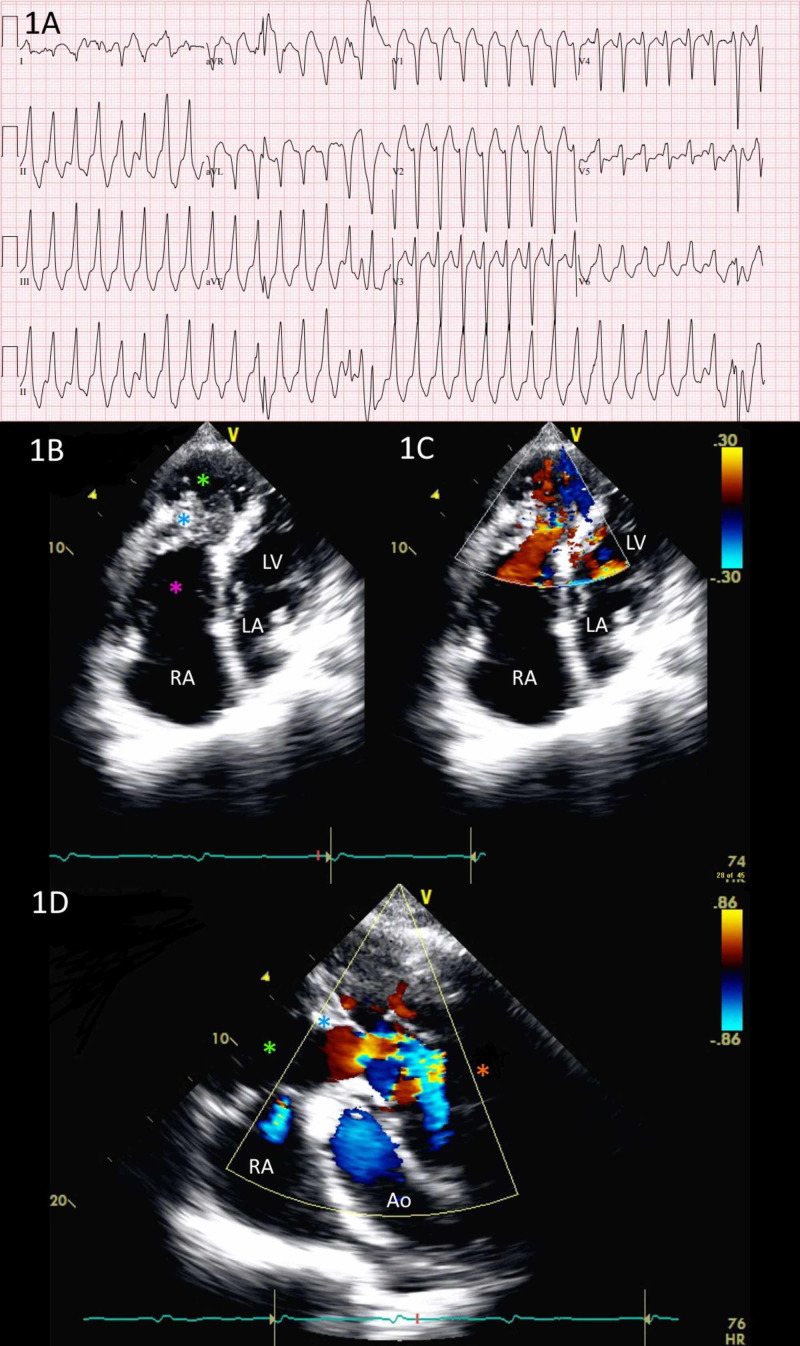
Electrocardiogram and echocardiogram data on diagnosing double-chambered right ventricle with ventricular tachycardia A) Electrocardiography (ECG) showing wide-complex tachycardia in a left bundle branch block pattern and inferior axis, consistent with RVOT-VT; B) Transthoracic echocardiogram, apical 4-chamber view. The anomalous muscular band (blue asterisk) that divides the RV into a distal chamber (green asterisk) and proximal chamber (pink asterisk) can be seen; C) Color Doppler showing turbulent mosaic-pattern blood flow through an opening in the muscular band; D) Short-axis view through the base of the heart. Color Doppler shows blood flow from the distal RV chamber (green asterisk) through an opening in the muscular band (blue asterisk) and into the RVOT (orange asterisk) RV: right ventricle; RVOT: right ventricular outflow tract; RVOT-VT: right ventricular outflow tract ventricular tachycardia

The patient is a 70-year-old Mandarin-speaking male with a past medical history of coronary artery disease, congenital heart disease, diabetes mellitus type 2, and other comorbidities who presented to the emergency department from his primary care doctor’s office with complaints of chest pain and palpitations. In the emergency room, he had a blood pressure of 82/30 mmHg and his heart rate was greater than 200 beats per minute. His initial electrocardiogram (EKG) showed a wide QRS with left bundle branch block morphology, inferior axis, and positive R wave starting in V4 that suggested right ventricular outflow tract ventricular tachycardia (RVOT-VT) that required external cardioversion (Figure 1A). After two cardioversions, his heart rate was converted to normal sinus rhythm. After the cardioversion, he complained of some chest discomfort and gastrointestinal upset. At that time, he reported that also he had an episode of chest pain the night before that resolved with nitroglycerin. Additionally, he mentioned being told of some heart condition at age 19 that was never fully evaluated and diagnosed; he refused surgery and had no symptoms until the current presentation. His pertinent cardiovascular medications were aspirin, 81 mg daily, atorvastatin, 10 mg daily, digoxin, 125 mcg daily, lisinopril, 2.5 mg daily, and spironolactone-hydrochlorothiazide, 25-25 mg daily.

He was started on intravenous amiodarone. Bedside transthoracic echocardiography revealed a left ventricular ejection fraction of 25% and two chambers were noted in the right ventricle, which was consistent with a DCRV (Figure 1B-D). He was diagnosed with obstructive coronary artery disease (80% lesion in the proximal left anterior descending artery) several years prior and refused revascularization at that time. The patient was switched to oral amiodarone but he experienced another episode of RVOT-VT, which resolved after another intravenous loading dose of amiodarone. He was found to have an elevated troponin. Surgical resection of the anomalous muscular band with possible coronary artery bypass grafting and subcutaneous implantable cardioverter-defibrillator (ICD) placement as treatment options were discussed. After much deliberation, the patient and his family initially refused all the options. Ultimately, the patient only agreed to the placement of a subcutaneous ICD. He was discharged home without further events during the hospital stay.

## Discussion

The presence of arrhythmias associated with DCRV is not commonly seen. One case of DCRV in adulthood coincided with wide complex tachycardia detected on Holter monitoring and the arrhythmia was improved after surgical intervention [3]. In the case of a 63-year-old female who complained of syncopal episodes and exertional dyspnea, she presented with sustained ventricular tachycardia (VT) in the setting of uncorrected DCRV [4]. Another report mentions two patients with DCRV who presented with monomorphic VT that was reproduced in an electrophysiological study and the VT for both patients resolved after surgery [5]. Some cases of arrhythmias after surgical correction of DCRV have also been reported. Selvaraj et al. reported a 35-year-old female having surgically corrected DCRV who presented with non-sustained VT due to a right ventricular apical scar that developed from the DCRV surgical repair [6]. One patient with DCRV was found to have postoperative paroxysmal atrial fibrillation with rapid ventricular response [7]. A 35-year-old male who had surgery at age 14 for DCRV presented with VT storm despite medical therapy and ICD therapy [8]. 

The standard management of DCRV begins with appropriate imaging to provide important presurgical information and is followed by surgical correction. We were able to correctly diagnose DCRV using transthoracic echocardiography, but we recognize that this may not be accurate enough for all patients. Therefore, diagnosing DCRV overall using echocardiography (transthoracic and/or transesophageal) with consideration of other diagnostic modalities, such as cardiac magnetic resonance imaging and cardiac catheterization based on need and institutional availability, may be best for patient care. After the appropriate diagnostic studies have been performed, surgical intervention is the primary treatment option recommended for patients with DCRV. Surgical correction is acutely recommended in nearly all cases with symptoms and associated features present or with an asymptomatic but significant obstruction with a pressure gradient > 40 mmHg [9]. Based on the report by Alvarez et al., the controlled induction of VT with an electrophysiological study followed by surgery and a repeat electrophysiological study to assess for postsurgically reproducible VT is an effective option [5]. Presurgical and postsurgical electrophysiological testing can be helpful to corroborate the effectiveness of surgery on removing any tissue associated with DCRV with the potential to be an arrhythmogenic origin. Surgery should be recommended with the best surgical approach being selected on a case-by-case basis based on presurgical information from appropriately selected diagnostic tests.

Options must also be considered if a patient presents atypically with associated arrhythmias. It is important to understand that ICD placement is useful for preventing sudden cardiac death, but it only reduces the burden of VT by terminating it instead of preventing it. Thus, medical therapy must be considered for the management of VT first. Antiarrhythmics, such as amiodarone, sotalol, and dofetilide, and non-antiarrhythmics, such as metoprolol, have been used. In a position paper on arrhythmias and congenital heart disease, electrical cardioversion for acute termination of hemodynamically stable or unstable VT was recommended while intravenous amiodarone or procainamide also was recommended if cardioversion could not be performed [10]. Additionally, this paper also recommended beta-blockers for non-sustained VT without increased risk for sudden cardiac death and antiarrhythmic agents as adjuncts to an ICD [10]. The Optimal Pharmacological Therapy in Cardioverter-Defibrillator Patients (OPTIC) trial randomized patients getting ICD shocks to a beta-blocker alone, sotalol, or amiodarone, plus a beta-blocker, and showed that the patients in the amiodarone, plus beta-blocker arm, received the lowest percentage of ICD therapy [11]. It is notable to also report that amiodarone had nearly an 18% discontinuation rate versus a 5.3% discontinuation rate for beta-blockers alone [11]. In the Antiarrhythmics Versus Implantable Defibrillators (AVID) trial, beta-blocker use did not improve survival in patients with ventricular fibrillation or VT who were already being treated with amiodarone or ICD therapy but it reduced mortality in patients not treated with amiodarone or ICD therapy [12]. Thus, amiodarone and beta-blocker use together did not have a synergistically positive impact on patient outcomes.

In our case of DCRV associated with preoperative VT, which, to our knowledge, has not been reported often in the literature, the patient refused surgery. However, he did agree to subcutaneous ICD placement, which we hoped would reduce the risk of sudden cardiac death secondary to fatal arrhythmias. Irrespective of the decision to consent to or refuse surgical correction, it is important to encourage proper follow-up in the outpatient setting and provide optimal long-term care for these patients.

## Conclusions

Adult patients who have lived with DCRV and remain undiagnosed until it presents later in life, as in our case, require appropriate cardiac imaging to diagnose this condition. Based on the aforementioned reports about DCRV patients, VT prior to surgical repair of DCRV is an uncommon presentation. In these cases of patients with a delayed presentation of DCRV in adulthood with associated VT who refuse surgery, the use of ICD therapy must be carefully considered on a case-by-case basis by accounting for the patient’s autonomy, comorbidities, medication tolerability and compliance, and long-term prognosis.
